# Maritime Telemedicine: Design and Development of an Advanced Healthcare System Called Marine Doctor

**DOI:** 10.3390/jpm12050832

**Published:** 2022-05-20

**Authors:** Gopi Battineni, Nalini Chintalapudi, Francesco Amenta

**Affiliations:** 1 Telemedicine and Telepharmacy Centre, School of Medicinal and Health Products Sciences, University of Camerino, 62032 Camerino, Italy; nalini.chintalapudi@unicam.it (N.C.); francesco.amenta@unicam.it (F.A.); 2 Research Department, International Radio Medical Centre (C.I.R.M.), 00144 Rome, Italy

**Keywords:** expert systems, seafarers health, ICT, Marine Doctor, desktop applications

## Abstract

*Background:* The availability of better healthcare services is critical for onboard seafarers. The development of expert systems can help ships with limited medical facilities, which allow the shipside doctors to properly refer symptoms to remote doctors. This allows clinicians to make a correct diagnosis from there, which leads to proper treatment. A software named Marine Doctor (M Doc) has been developed by incorporating computing technologies to address this objective. *Methods:* With the help of Information and Communication Technology (ICT) this application can support the provision of appropriate medical assistance to seafarers. The system was developed with Python Tkinter (frontend) and PHP (backend) languages. MySQL was used as a server database. *Results:* Seafarers can use M Doc to benefit from medical advice that can reduce complications due to misdiagnosis and help doctors to make better-informed decisions. By automatically collecting appropriate sequences of symptoms, doctors will be able to generate proper information for referral of patient symptoms and subsequent advice based on the data. *Conclusions:* Technology that supports experts on board ships in better interacting with Telemedical Maritime Assistance Services (TMAS) could define the future of medical assistance at sea.

## 1. Introduction

Due to diseases or accidents, it has always been a challenge to provide adequate healthcare to those who work on ships [1]. Seafarers’ lack of medical staff on board, their low level of medical knowledge, and the limited availability of medical supplies place them in a disadvantageous position compared to those living ashore [2]. In the event of diseases or accidents, individuals on board ships will try to arrange treatment for themselves or, in more severe cases, will seek the advice of a Telemedical Maritime Assistance Service (TMAS) or the intervention of rescue media to bring sick or traumatized (MEDEVAC) persons ashore [3,4].

The appropriate use of modern communication and remote medical technology is crucial for seafarers. There has been a growing interest in the field of Information and Communication Technology (ICT), and expert systems are among the fastest-growing technologies [5,6]. A computer program composed of a set of production rules based on knowledge obtained from an expert source represents the knowledge obtained from a human expert. This knowledge can be used to solve complex problems. An expert system is a computer program that replicates the behavior of an expert human. Since these systems use human expert knowledge to solve complex problems in many areas, they have an impact on many areas of our lives.

It is difficult for ship captains or officers charged with on board medical assistance to describe the symptoms or injuries of seafarers simply due to their limited medical knowledge. As a result of this challenge, the TMAS doctor will ask several questions to arrive at a presumptive diagnosis, which will determine the appropriate treatment of the problem(s) [7]. A TMAS doctor can now conduct an assessment in person without being on board due to advancements in technology. To do so, digital devices are used. These devices can record vital signs; monitor progress; view external lesions; take photos of skin, ears, and eyes, among other things; and take telemedicine to another level [8].

A realistic possibility to obtain medical care for patients located in remote sites such as seagoing vessels, in which health professionals are not available, is to contact a TMAS doctor via telecommunication systems [9]. Seafarers are always in need of a first-level telemedical consultation because the medical knowledge of onboard personnel in charge is not good enough to treat the patient [10]. To maintain correct information flow an upgraded technological capability will be required for TMAS centers that are driven to offer a software platform that is accessible to everyone on board. Therefore, the development of an expert system helps to transmit the correct diagnosis of the most common pathologies for seafarers [11]. These systems described here allow people responsible for onboard medical care to forward detailed requests for assistance that contain symptom-guided information on patient clinical conditions. This may represent an innovative tool for medical consultations at a distance, allowing the remote center to provide quicker and more precise medical advice.

Telemedicine at sea is regarded by the International Maritime Organization (IMO) as an essential part of rescue procedures [12]. The captain, or their representative, may contact the TMAS if onboard pathologies or accidents require medical intervention when there is no onboard medical or paramedical staff. Although there is some personnel on board who have completed first aid or healthcare training, these individuals have very limited medical capabilities. As a result, except for responsible auto-medication, it is unlikely that clinical initiatives are taken without the expertise of TMAS physicians. Traditionally, requesting medical assistance from a ship to an offshore medical center follows the same procedure as it did 100 years ago [13].

Our technical team at International Radio Medical Centre (C.I.R.M) has developed a telemedicine system called Marine Doctor (M Doc) that works as an easy-to-use software to assist crew members who have no medical background in properly interacting with TMAS physicians. Compared to the conventional consultation systems based on telephone and e-mail, the proposed device is more accurate and complete in terms of information contained in the request for assistance.

Moreover, data received by the medical center can be more easily managed, as they can be standardized. The C.I.R.M service center currently maintains a high-speed internet connection, web server, and the latest software to handle previous patient data. M Doc works as an intelligent guide to collect symptoms and can be used by ship captains in the event of medical problems or emergencies that are commonly encountered on board. In addition, with this app, healthcare providers are able to assess, diagnose, and treat seafarers without having to visit them personally. Onboard, the passengers can communicate with TMAS physicians through this dedicated telehealth expert system. Here, we present the development of the M Doc, where TMAS doctors collect and analyze all symptomatic information about onboard medical problems.

## 2. Methods

The major functionality of the M Doc application is based on whether an incident was a sickness or an accident on board. Application development has been divided into different steps, including analyzing medical records, designing, coding, testing, and evaluating.

### 2.1. Data Collection

After a discussion with doctors at the Italian TMAS center called C.I.R.M, we drafted ideas to analyze the medical records of seafarers’ on-board incidents. We concluded that a detailed explanation regarding seafarers’ data (name, rank, country, and age), basic biomedical information (height, weight, pulse rate, blood pressure, temperature, glucose, and oxygen levels), incident description (how, when, and where it happened), symptomatic information, and medical notes (prescriptions, medicines, allergies, etc.) are the minimum required pieces of information to transmit to onshore doctors for better diagnosis.

### 2.2. Software and Hardware Requirements

✓ Programming languages: Python was used to develop the Graphical User Interface (GUI) and the ‘T-kinter’ package to interface with the GUI toolkit [14]. The backend was developed in PHP.
✓ Databases: Two databases were made available for application functionality: a MySQL database generated in the cloud and a SQL lite database that functions locally.
✓ Hardware: The system works on any PC with 64-bit Windows versions.
✓ For data storage, Windows server 2019 was used.

### 2.3. Designing and Functionality

**Figure 1:**
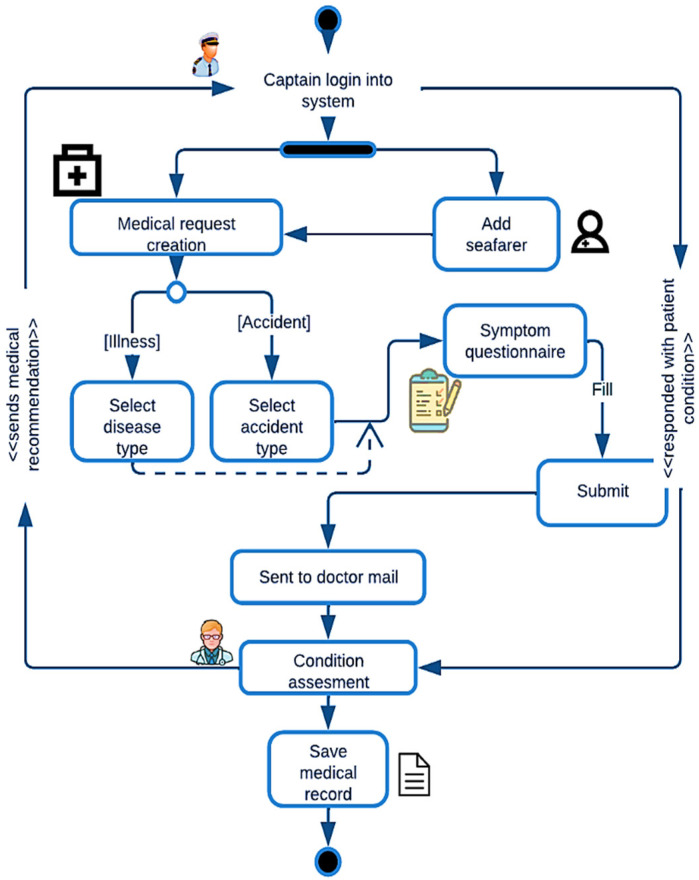
M Doc activity diagram.

The design and architecture were created based on this knowledge base [15]. In software development, the design phase serves as input for the development phase. The next step involves designing roadmaps, sketches, workflow charts, Unified Modelling Language (UML) diagrams [16], and prototypes. In these designs, the project supervisor incorporated insight analysis system specifications. Security risks were identified, assessed, and sent for approval. The development followed after requirements and specifications were complete. The step-by-step application functionality can be seen in Figure 1.

### 2.4. Data Protection

This application is committed to maintaining the confidentiality and protection of seafarers’ data respecting the General Data Protection Regulation (GDPR) [17]. Patient data are collected to provide direct healthcare services. However, if required by law, this information can be disclosed with consent or when justification as to the public interest can be demonstrated. The public interest includes the safety of the other crew on board. Our effort to provide health statistics and trends will involve using persons’ medical data anonymously. The data may include demographic data, such as date of birth, and patient health information that is recorded in coded form.

## 3. Results

The purpose of the M Doc system is to simplify the medical assistance of seafarers and to develop software that helps patients of seafarer groups. The present software is friendly, simple, fast, and compatible with any computer. In contrast to traditional methods of data collection, M Doc has the advantage of providing insight into patient symptoms. Seafarer and doctor details are registered and stored by the system, and a simple questionnaire guides users through the process of sending medical requests. Below are the procedures of how to comply with data protection laws and ensure the process is confidential. In addition to secure login, shipping companies have access to a list of ships for which they have subscribed to the service. The data will be further stored securely in a windows server that is accessed by VPN authentication with SSL certification [18].

### 3.1. Features of the M Doc

- It automatically sorts incoming medical requests from the vessel and is a secure system with all required data protection protocols and settings to comply with required regulations.
- Once the vessel is automatically sorted, the medical list (based on the flag state and information provided) is visible to the doctor on duty.
- The medical request questionnaire form serves as a guideline for captains to ensure all relevant information is captured in the first instance.
- This helps quicken the response time. The seafarer requiring medical assistance is easily identified by using the ship ID, which is captured in the medical request.
- All correspondence on the medical event is captured in the system.
- All medical events are classified by WHO–ICD 10 codes, which provide the basis for analysis and health prevention initiatives later.
- This system is fully integrated with a sophisticated telemedicine case from the testing ships.

### 3.2. System Functionality

#### 3.2.1. Home Page and Menu Icons

**Figure 2:**
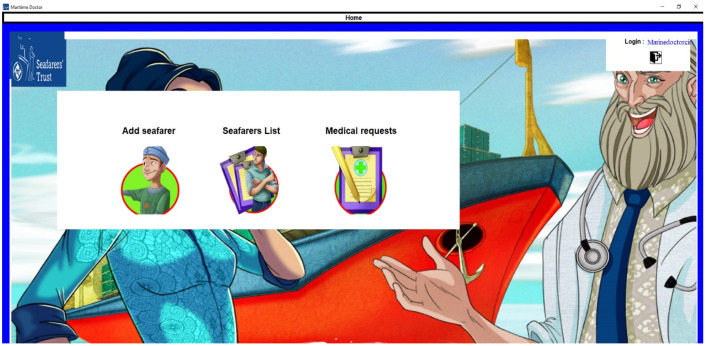
Application home page.

The captain or medical officer on board is the one who logs into the registration system. As soon as the captain logs into the software, the screen will display three icons namely add seafarer, seafarers list, and medical request, which can be visualized in Figure 2.

#### 3.2.2. Seafarers’ Registration

**Figure 3:**
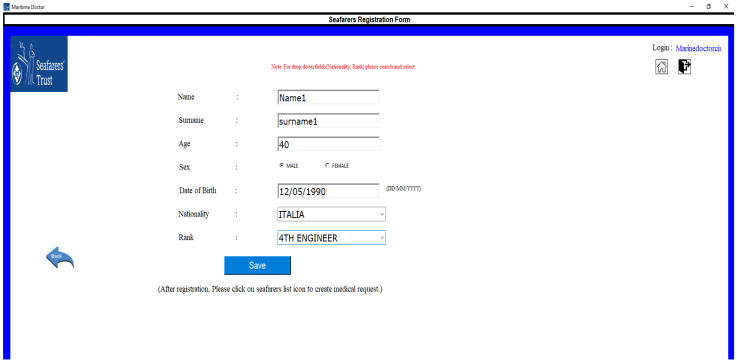
Seafarer registration form.

By clicking on add seafarer, the system will prompt the captain to fill out the seafarer details form (Figure 3). The added seafarer data will be stored in the application and when they become ill or injured, the captain initiates medical requests to the doctor.

#### 3.2.3. Ship Position and Vital Symptom Data Collection

**Figure 4:**
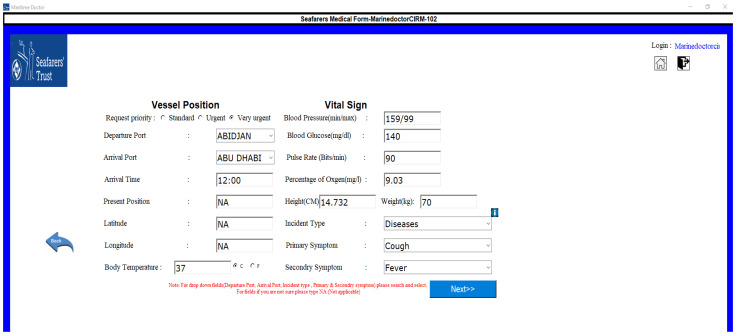
Form to collect ship location and patient biomedical information.

Once the medical request has been initiated, the ship location and vital symptoms of the patient will be collected by the form shown in Figure 4.

#### 3.2.4. Disease Questionnaire and First Aid Display

**Figure 5:**
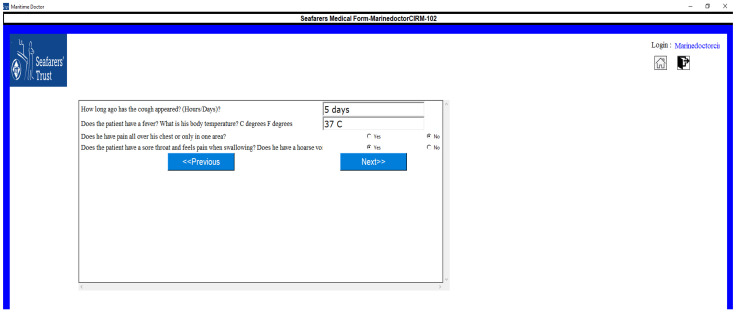
Display of symptomatic questionnaire.

**Figure 6:**
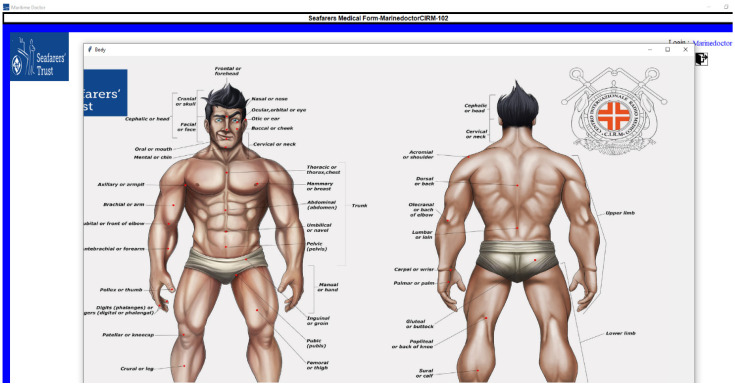
Representation of anatomical terminology.

**Figure 7:**
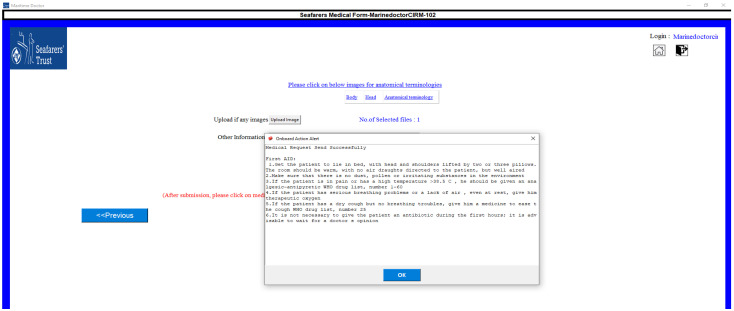
First aid display.

The symptomatic questionnaire related to a particular disease will appear in the follow-up screen, which can be observed in Figure 5. There are some situations where a captain does not have any experience with anatomical terminology, so we have provided a detailed descriptive view of anatomical terminology, which is presented in Figure 6. Once all the questionnaires are completed, a medical request will be generated, and once the submit button is clicked, the system will display the first aid action for that particular illness (Figure 7).

#### 3.2.5. Doctor Response

**Figure 8:**
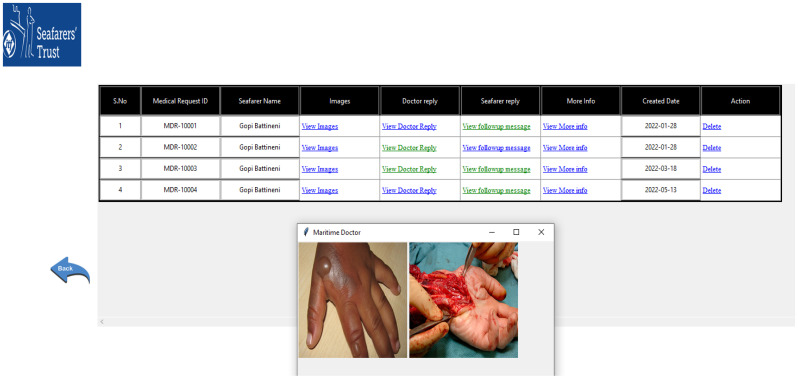
Onboard doctor response.

Based on the information provided, the generated medical request is delivered to the onshore doctor to assess the patient’s condition. Once the medical request has been transferred to the doctor’s mail, the doctor will assess the symptomatic information and suggest necessary prevention measures. The uploaded images and doctor replay will appear in the software. By using color coding, the captain will be able to recognize that read messages are green, while unread messages are blue (refer to Figure 8).

## 4. Discussion

The quality of medical care that can be provided onboard will increase without a doubt as a result of telemedicine systems. Although technology has progressed, low-quality medical care can still be provided onboard ships due to limited medical competency on board [19,20]. Based on actual cases of assistance, an intelligent guide could be useful in aiding sailors in the preparation of a medical request. It is helpful for the remote doctor to have these details and information on hand when treating a disease or injury onboard since it will allow them to provide the best care for the patient [19,21].

The main objective of any TMAS center is to provide better health to seafarers with no time elapse [3]. Due to the great demand for supplemental health services for seafarers, they are always trying to make it better and better every day. However, by conventional communication, it is hard to give prompt and effective responses to patients as quite often requests for medical advice from ships lack basic information to allow for correct diagnosis and identification of diseases/illnesses affecting seafarers [22].

Our goal is to identify potential for health improvements and make accessing health services viable for every patient on ships around the world. Our expert team and research team have been working together for many years to achieve these goals. The result shall be the design of data mining technologies for patient health improvement. This is of particular importance as the number of people requiring medical advice to C.I.R.M is increasing every year, and to guarantee the quality of the service, we need to identify problems affecting seafarers quickly and we should provide our medical assistance as soon as possible.

This M Doc software is intended to be used as a desktop application for seafarers’ health management [23]. Usually, desktop applications run on desktop and laptop computers independently of other applications unlike a Web-based application, which is accessible via a Web browser. Initializing the internet after installation of this type of software is not required. Computer desktops are used for installing the software. Later, it can be accessed by a specific username and password provided. Furthermore, these details are retrievable when required and can be manipulated meaningfully. System inputs contain information about the patient and the diagnosis, whereas system outputs display the data. Data can be added to the database either by an administrator or by crew members.

It can be argued that a potential problem would be the limited usage of phones on board ships due to the extreme danger of using a cell phone while wearing a navigational watch. A vessel’s bridge is the place with the greatest coverage of cell phone signals at sea, and in many cases, the master is equipped with a phone that is approved by the shipping organization to ensure seamless communication between ship and office [22]. Because of poor satellite signals, the voice breakdown and sometimes severe weather can interrupt the services on the sea [22,24].

There is some existing software from our center that was developed to maintain a ship’s pharmacy [25], manage the medicine chest [6], maintain seafarers’ health passports and patient health records [26], and record the seafarer’s physical activity through android-based smartphone application [27]. However, these applications are limited to their functionality and are not for the transfer of telemedical requests to TMAS doctors. We are considering a move to create and provide an online platform by using ICT technologies for better accessibility of medical assistance to seafarers.

Till now, C.I.R.M has only provided its services through e-mail/telephone. Once the person in C.I.R.M has received the mail or call from the seafarer, then he/she will connect this to the corresponding specialist. The concerned doctor will respond based on their free time, which means that it would take more time than expected to provide medical service. Because of this, C.I.R.M has not been able to provide accurate information to our customers within time. By offering advanced telemedicine services, it is possible to expand services entirely to new technologies and growth projections and allow seafarers to monitor their health issues from the convenience of onboard ships. There are no proposed changes to C.I.R.M’s current services offered because of this study as these services are free of cost.

By considering all these factors, C.I.R.M. considers the M Doc application as a dedicated application to record, manage, and track the medical requests received from vessels. This application integrates with the other applications to provide a seamlessly connected healthcare framework for seafarers. At the time of a medical request, a minimum medical data referral form can be used by the captain to capture the basic information required for the doctor to make an informed medical recommendation. To guarantee the best assistance, the use of the M Doc software as guidance in requiring medical advice is recommended. Moreover, the availability of ships for system testing allows for the transmission of biomedical data, guaranteeing the delivery of high-quality distance medical assistance.

Despite its advantages, the M Doc application has its limitations. Primarily, the present system is not compatible with PC with low operating system configurations (32-bit). Similarly, in-person visits are not a complete substitute for telemedicine expert systems, and they may not apply to every situation [28]. For example, this system does not include smartphone-based 3D scanning that shows the technical difficulties that are integrated with care delivery. In addition, this application lacks video calling support because the system should have an 8 Mbps internet connection, which is unlikely on cargo ships. These issues will be addressed in future updates.

## 5. Conclusions

In this paper, we presented the core knowledge behind M Doc development and its workflow. We explained the ideas behind the conceptualization of developed software and recently updated it based on user requirements to improve its efficiency. This system can be well-responded to with some predefined medical requests and can successfully present help in terms of immediate first aid and medical prescriptions by an onshore doctor. These advanced telemedicine systems are enabling surgeons to make better prescriptions after viewing digital images of some serious issues such as burns and open wounds. The purpose of medical expert systems is, therefore, to provide valid prevention characteristics for seafarers’ health and to make new dimensions in the maritime industry.

## Data Availability

Not applicable.
